# Role of coherence and delocalization in photo-induced electron transfer at organic interfaces

**DOI:** 10.1038/srep32914

**Published:** 2016-09-08

**Authors:** V. Abramavicius, V. Pranculis, A. Melianas, O. Inganäs, V. Gulbinas, D. Abramavicius

**Affiliations:** 1 Vilnius University, Faculty of Physics, Department of Theoretical Physics, Saulėtekio 9, LT-10222 Vilnius Lithuania; 2 Center for Physical Sciences and Technology, Savanoriu 231, LT-02300 Vilnius, Lithuania; 3 Biomolecular and Organic Electronics, Department of Physics, Chemistry and Biology (IFM), Linköping University, SE-581 83 Linköping, Sweden

## Abstract

Photo-induced charge transfer at molecular heterojunctions has gained particular interest due to the development of organic solar cells (OSC) based on blends of electron donating and accepting materials. While charge transfer between donor and acceptor molecules can be described by Marcus theory, additional carrier delocalization and coherent propagation might play the dominant role. Here, we describe ultrafast charge separation at the interface of a conjugated polymer and an aggregate of the fullerene derivative PCBM using the stochastic Schrödinger equation (SSE) and reveal the complex time evolution of electron transfer, mediated by electronic coherence and delocalization. By fitting the model to ultrafast charge separation experiments, we estimate the extent of electron delocalization and establish the transition from coherent electron propagation to incoherent hopping. Our results indicate that even a relatively weak coupling between PCBM molecules is sufficient to facilitate electron delocalization and efficient charge separation at organic interfaces.

Absorption of sunlight in OSCs primarily creates neutral molecular excitations, while their conversion to electron-hole (e-h) pairs occurs at the heterojunction between electron donating (polymers or small molecules) and electron accepting (fullerenes or their derivatives) materials. Electron-hole pairs split into free charge carriers on a femtosecond time scale and despite the strong Coulomb interaction between the charges this process is known to be often close to 100% efficient. Although several often conflicting models have been proposed, to this day it remains unclear what mechanism is responsible for efficient charge separation at organic heterojunctions. Several explanations were suggested for this initial dissociation stage, for example: charge carrier delocalization over several polymer segments and/or fullerene molecules^1^, hot interfacial charge-transfer (CT) states with delocalized wave functions^2^,^3^, or, alternatively, with electron and/or hole wavefunctions localized on molecules situated at large distances from the interface^4^. Recently, a partially coherent model, assuming electron delocalization over the entire aggregated fullerene domain^5^, and a hybrid model of a 1D polymer/fullerene lattice with semi-classical dynamics at short time scales and Redfield relaxation theory at long time scales^6^, have been proposed. Consequently, charge separation on an ultrafast time scale is often considered to be predominantly coherent. However, carrier delocalization and coherent propagation, their extent and temporal evolution have only been qualitatively postulated. Although the later time scales of charge separation have been successfully described by incoherent hopping^7^, a consistent model at the earliest time scales, describing the coherent propagation of charge and the gradual transition into the classical hopping regime, is still absent. As a consequence, the role of coherence remains elusive.

To elucidate the role of delocalization and coherence in charge separation a full quantum mechanical description is necessary. Here, we use the stochastic Schrödinger equation (SSE) to describe the charge separation process by the principles of open quantum systems. This approach allows us to evaluate the interplay between coherent dynamics and bath induced dephasing^8^,^9^, and predict the importance of delocalization in systems with a wide range of intermolecular couplings, leading to different charge separation scenarios. Our model predictions are consistent with experimentally measured charge carrier separation dynamics on an ultrafast time scale, as confirmed by a recently developed experimental technique^10^. This article thus reveals the role of delocalization and coherence in aiding charge separation at organic interfaces.

We consider charge separation at the heterojunction of a conjugated polymer and an aggregate of the fullerene derivative PCBM. Our results are also applicable to small molecule donors and pristine fullerenes. It has been recently experimentally demonstrated that mainly electron motion in PCBM is responsible for the initial evolution of the charge separation process – hole motion is significantly slower^5^,^11^,^12^. Therefore, in our treatment we consider the hole to be immobile. Consequently, we construct a finite cubic lattice of acceptors and a single donor site for the hole (see the Methods section for details and a schematic). The variable size of acceptor aggregates and the overall complexity of interfaces in the hierarchical morphology of the bulk heterojunction blend is reflected by a variation of couplings between the acceptor sites. In order to model photo-excitation in the donor phase, we initially place both the electron and the hole at the donor site and let the electron propagate in the electrostatic field of the stationary hole. We set simulation parameters typical for polymer-PCBM solar cells (see Supplementary Information), however, we find that the most important parameter governing the coherent (<500 fs) dynamics of the electron is the inter-acceptor coupling *J*_A_. Variations of other parameters: donor site excitation energy *ε*_D_, CT state energy *ε*_CT_, donor-acceptor interaction energy *J*_DA_ and system-bath interaction energy *λ* only slightly affect the evolution of the system – while the dynamics at later times (>500 fs) somewhat differ, they remain qualitatively the same, see Supplementary Information. Hence, in most of our analysis we keep the parameters fixed, except for the inter-acceptor coupling *J*_A_, which was varied. We find that within these ranges our model gives quite a narrow spectrum of possible charge separation scenarios. All of them lead to charge separation kinetics which qualitatively agree with experimental results. This means that the simulation results presented below are suggestive of the underlying physical phenomena occurring in operating organic solar cells.

Figure 1 shows the simulated temporal evolution of electron density for the indicated values of inter-acceptor electron coupling *J*_A_. Data are projected in the plane perpendicular to the donor/acceptor interface. External electric field is not applied to highlight the effects of delocalization. Our results show that the electron is transferred from the donor site to a nearby pool of coherently coupled acceptor sites within ~300 fs. The number of accessible sites in a given time interval grows with increasing intermolecular (inter-acceptor) coupling, allowing for the electron to transfer to more distant sites already at very early times. Electron transfer is quantitatively characterized in the rightmost column of Fig. 1, where the kinetics of the absolute e-h separation distance, *d*_*abs*_(*t*) (Fig. 1 black traces) and the electron delocalization radius, *l*(*t*) (Fig. 1, blue traces), are shown (details on these parameters are given in the Methods section). The extent of electron delocalization is visualized by the blue circles. These results only weakly depend on the donor excitation energy *ε*_D_ (see Supplementary Information), in agreement with experimental studies of charge separation efficiency versus excitation energy^13^.

The kinetics of both *d*_*abs*_(*t*) and *l*(*t*), for different *J*_A_ exhibit a similarly rapid initial rise. For very weak coupling both electron delocalization and initial electron transfer distance are small, and thus the electron is only transferred from the donor to the nearest acceptor site. For intermediate coupling the initial transfer distance is increased and the electron is more, although still weakly, delocalized. In this regime at later times >500 fs the average electron distance increases while its delocalization remains constant. In case of strong coupling, the electron is strongly delocalized, we thus observe the largest electron transfer distance at early times, whereas the later part of the transfer process is mainly determined by time-dependent localization. Note that for strong couplings electron delocalization is confined by the size of the acceptor lattice in our model, chosen to correspond to the expected PCBM domain size (8 × 16 × 16 nm^3^) in efficient polymer-PCBM blends (see the Methods section). Despite similar probability distributions at long times in the second and third rows of Fig. 1, the extent of delocalization of individual electrons and, consequently, the character of their motion is very different.

To determine which regime corresponds to real polymer-PCBM blends, we have compared our model prediction to experimentally measured carrier drift dynamics probed by a recently developed experimental technique, time-resolved electric-field induced second harmonic generation (TREFISH), enabling ultrafast measurements. We have chosen a bulk heterojunction solar cell based on P3TI-PC_71_BM. This system is of particular interest due its exceptionally low driving force Δ*E* ~ 0.1 eV for charge separation^14^, nevertheless leading to an internal quantum efficiency (IQE) of 90%. Transient absorption spectroscopy also indicates ultrafast photo-induced charge transfer on a <100 fs time scale, see Supplementary Information. Charge separation in P3TI-PC_71_BM is thus expected to rely on charge delocalization and coherent electron propagation.

Figure 2 shows the experimental and simulated average e–h separation along the direction of the electric field of 5.7 × 10^5^ V/cm, created by applied voltage and the built-in field of the OSC (see the Methods section and the Supplementary Information for a more detailed description). Using transient absorption spectroscopy we observe no signatures of delayed charge transfer due to exciton diffusion, only prompt photo-induced charge transfer at a time scale of <100 fs, see Supplementary Information. Thus, the experimental EFISH data in Fig. 2 directly monitors the motion of the photo-induced electron away from the donor/acceptor interface. The experiment shows a fast ≈500 fs initial rise to a charge separation distance of 0.4 nm. The later part of the charge separation process is considerably slower – separation distance gradually rises up to 0.9 nm in 3.5 ps.

To simulate the experiment we have added an external electric field to the model and have also taken into account the fact that donor-acceptor interfaces can be arbitrarily oriented with respect to the direction of the field. We reproduce this situation by performing simulations with a randomly oriented electric field and averaging the obtained e-h separation distance projected along the direction of the field. Our model with *J*_A_ = 12.5 meV (intermediate coupling) reproduces the experiment (see Fig. 2) and points to an absolute carrier separation distance of 2.5 nm in 500 fs as shown in Fig. 1. It is considerably smaller than the 4 nm distance evaluated in Ref. 5 for PCDTBT:PC_61_BM. The latter corresponds to our model prediction in the strong coupling regime where we also obtain a carrier separation distance of 4 nm (Fig. 1c). However, at least for the case of P3TI-PC_71_BM, comparison with experiment indicates that such coupling and initial separation are overestimated. Given that P3TI-PC_71_BM operates at an IQE of 90%, we thus suggest that intermediate couplings, leading to electron delocalization just over two lattice sites (Fig. 1), are already sufficient to facilitate efficient charge separation at organic interfaces.

To further support our results we calculate electron mobilities using the same set of parameters. The obtained values are 0.03, 0.37 and 1.26 cm^2^V^−1^s^−1^ for *J*_A_ = 1, 12.5 and 31.5 meV respectively. The predicted mobility at *J*_A_ = 12.5 meV is in excellent agreement with the experimentally measured electron mobility value of 0.3 cm^2^V^−1^s^−1^ in PCBM at the picosecond time scale^15^.

Classical hopping models have been used extensively to describe electron-hole separation and subsequent charge motion in organic materials^7^. The simulations rely on the initial e-h distance distribution following photoexcitation, which is difficult to access experimentally. Initial charge separation distances of 3–4 nm have been estimated^16^. Our present simulations allows us to visualize the formation of the e-h distance distribution with high spatio-temporal resolution. We find that coherent electron transfer lasts up to ~500 fs and is responsible for shaping the “initial” electron-hole distance distribution. Following the coherent propagation stage charge separation kinetics gradually switch to the slower phase (see e.g. the rightmost column of Fig. 1b where the *d*_*abs*_(*t*) trace features a two-phase evolution), which effectively corresponds to the incoherent-hopping phase. Thus, 500 fs marks the transition from coherent electron propagation to incoherent hopping where classical hopping models become valid.

Figure 3 shows the temporal evolution of e-h distance distribution without external electric field in the intermediate coupling regime. The electron gets completely transferred from the donor site to the acceptor lattice in ~500 fs, however, it is still strongly bound to the hole. Further charge separation is facilitated by incoherent electron hopping as outlined in ref. 17. The resulting e-h distance distribution at 800 fs can be approximated as an exponential (red dashed line in Fig. 3) and could be used in classical hopping models as the initial distribution.

Before we conclude, we have to point out that our model does not include geminate recombination, which although considered insignificant in efficient OSC systems, is necessary for a complete description. The extent of electron delocalization, as elucidated here, may be one of the key factors minimizing geminate recombination^18^.

In conclusion, our results suggest a complex coherence dynamics and their role in photo-induced electron transfer at organic interfaces: electron delocalization occurs on a femtosecond time scale, during which the electron wavefunction spreads in the acceptor phase. Although the extent of the electron wavefunction in PCBM is limited to only several molecules, it is already sufficient to facilitate an average electron-hole separation distance of ~2.5 nm on a femtosecond time scale. Coherent propagation also shapes the “initial” e-h distance distribution, which can be implemented in classical hopping models that are valid at time scales >500 fs – the transition time from coherent propagation to incoherent hopping. Finally, it should be noted that our proposed model is not specific to OSCs and may be generalized to explain charge transfer not only at any molecular interface but also in other collectively coupled molecular systems.

## Methods

### Experimental

#### Sample preparation

A monolayer of poly(3,30-([(90,90-dioctyl-9H,90H-[2,20-bifluorene]-9,9-diyl)bis(4,1-phenylene)]bis(oxy))bis(N,N-dimethylpropan-1-amine)) (PFPA-1) interface material was deposited on ITO coated glass substrates that were TL-1 treated. Poly[N,N′-bis(2-hexyldecyl)isoindigo-6,6′-diyl-alt-thiophene-2,5-diyl] (P3TI) and [6,6]-phenyl-C71-butyric acid methyl ester (PC_71_BM) (D/A 2:3 w/w) were then spin-coated from a 20 g/l o-dichlorobenzene (ODCB) solution with 2.5% (vol%) of 1,8-Diiodooctane (DIO). The thickness of the active layer (~70 nm +/− 10 nm) was determined by a Dektak surface profilometer. PEDOT:PSS PH1000 with a layer thickness of 110 nm was deposited from an aqueous solution mixed with 5% (vol%) of dimethyl sulfoxide and 0.5% (vol%) Zonyl FS 300 as the surfactant, and then annealed at 60C to remove residual water. The device was then encapsulated by a glass lid.

#### Experiment

**Figure 2:**
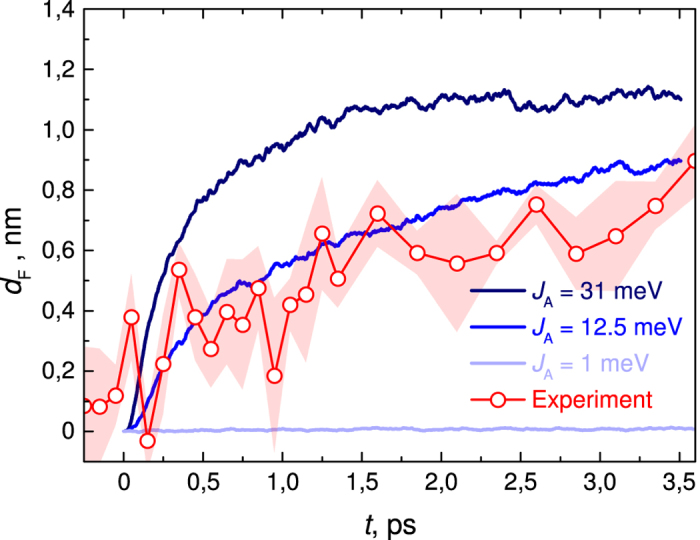
Time dependence of the charge separation distance *d*_*F*_ along the direction of the electric field *F* of 5.7 × 10^5^ V/cm for the indicated inter-acceptor coupling values *J*_A_. Symbols - experiment, shaded area – standard error in experiment, solid colored lines – simulations at different values of *J*_A_.

The time-resolved electric-field-induced second harmonic generation (TREFISH) technique has been described elsewhere^10^. Briefly, we use the Electric Field-Induced Second Harmonic (EFISH) generation effect to monitor the temporal evolution of the electric field strength in the bulk of the OSC active layer. Photogenerated charge carriers partially screen the applied electric field, leading to a reduction of the 2^nd^ harmonic intensity and allowing for their motion to be evaluated optically with sub-picosecond time resolution. We have used 130 fs, 800 nm, 1 kHz repetition rate pulses, generated by a Ti-Sapphire laser (Quantronix Integra-C) to excite the blend biphotonically. A fraction of the same laser pulse was used as the probe for the second harmonic (at 400 nm) intensity in the active layer. The 2^nd^ harmonic intensity transient can be related to changes in electric field strength in the active layer by I_2h_ ~ E^2 ^10. Electric field dynamics with sub-picosecond time resolution were obtained by varying the time delay between the excitation and the probe pulse, which was achieved by a mechanical delay stage. The intensity of the pump was set such that the electric field change caused by the optical excitation itself was significantly smaller than the electric field created by external voltage and the built-in field of the OSC. To avoid sample degradation external voltage was applied by reverse bias pulses of 10 μs duration, synchronized to those of the laser pump. The carrier separation distance along the direction of the electric field was determined as described in ref. 7. Standard error in experiment (Shaded area in Fig. 2.) was estimated as 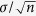, where *σ* is the standard deviation and *n* is the number of collected data points at a particular pump-probe time delay.

### Simulations

We describe the acceptor medium with the donor site as a quantum system characterized by the lattice Hamiltonian


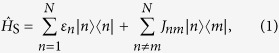


where *ε*_*n*_ is the electron energy on the *n*-th site and *J*_*nm*_ is the electron hopping energy between sites *n* and *m* (only one-particle states are included); state |*n*〉 denotes the electron on site *n*. The environment (heat bath) consists of harmonic oscillators (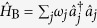). Here *ω*_*j*_ is the frequency of the *j*-th oscillator, 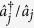 are the bosonic creation/annihilation operators of *j*-th bath mode and *ℏ* = 1. We assume only linear coupling between the system and the environment characterized by the interaction Hamiltonian 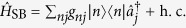). Here *g*_*nj*_ are constants describing the coupling strength between the *n*-th site and the *j*-th bath oscillator.

The heat bath consists of an infinite number of oscillators whose distribution is described by spectral density *C*^′′^(*ω*) = ∑_*j*_|*g*_*nj*_|^2^*δ*(*ω* − *ω*_*j*_). We use the environment model based on the Debye spectral density which has the form of an overdamped Lorentzian^9^.

**Figure 4:**
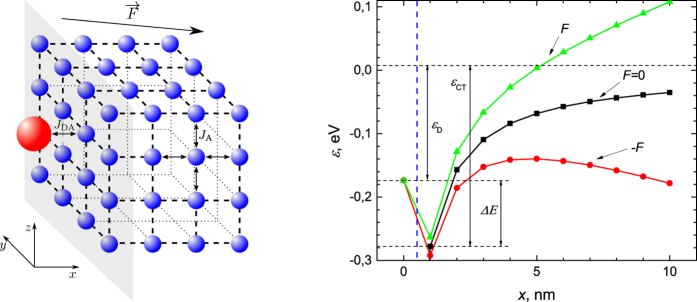
Model description. (**a**) Schematic of the donor - acceptor interface model. Red sphere denotes the donor site; blue spheres denote the acceptor sites; ***F*** denotes the arbitrary direction of the external electric field reflecting the averaging procedure over all possible CT interface orientations with respect to ***F***, see the main text; *J*_DA_ and *J*_A_ denote the interaction energies between the donor and the nearest acceptor site and between the nearest neighbor acceptor sites respectively. (**b**) Energy profile in the direction perpendicular to the donor/acceptor interface plane (dashed blue line), parameter values are given in the Supplementary Information. The red and green curves represent the shift of the Coulomb potential due to the applied external electric field 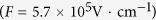, the black curve - the shifted Coulomb potential when a net zero electric field is applied. The donor site corresponds to *x* = 0. *ε*_D_ is the donor site excitation energy, *ε*_CT_ denotes the state energy of the CT exciton and Δ*E* is the driving force.

The model is implemented on a 3D cubic lattice of 8 × 16 × 16 sites (Fig. 4 (a)). This lattice size corresponds to the expected size of a PCBM aggregate in well-intermixed bulk heterojunction solar cells. Transmission electron microscopy images of P3TI:PC_71_BM^14^ indicate a well-intermixed morphology, in agreement with our assumption. Each site of the acceptor (molecule) can be free or occupied by an electron. The CT state is obtained when the electron is transferred from the donor to the neighboring acceptor site. At all times the donor site remains occupied by a hole, i.e., the donor site is merely the source of the electrostatic Coulomb field and the electron is described quantum mechanically using the SSE.

Site energies *ε*_*n*_ in the Hamiltonian 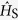 (Eq. 1) are given by


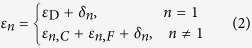


Here *ε*_D_ is the electron self-energy on the donor site (it is the molecular excitation energy since the hole is localized on this site as well), while self-energies of the acceptor sites are zero, *ε*_*n*,*C*_ is the Coulomb interaction energy between the hole and the electron, *ε*_*n*,*F*_ is the energy contribution due to some external electric field and *δ*_*n*_ - the contribution due to the static energetic disorder of electron energies in the lattice. *δ*_*n*_ is taken as a Gaussian random number, characterized by the standard deviation *σ*, and the results must be averaged over this parameter. Interaction energy *J*_*nm*_ in the system Hamiltonian is equal to *J*_DA_ if *n* and *m* denote the donor and the nearest-neighbor acceptor site, otherwise, *J*_*nm*_ = *J*_A_ if *n* and *m* denote nearest-neighbor acceptor sites.

Coulomb potential between the hole and the electron has the form 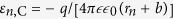, where *q* is the electron charge, *∈*_0_ and *∈* are the dielectric constants of empty-space and of material, respectively, *r*_*n*_ is the distance between the *n*-th site of the lattice and the donor site, parameter *b* accounts for the finite size of the donor and acceptor molecules shifting up the CT exciton energy according to the experimentally determined binding energy. The energy term 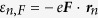 is due to the external electric field ***F***. Using Eq. 2 and expressions for *ε*_*n*,C_ and *ε*_*n*,*F*_ we can visualize the energy profile in the *x* direction, perpendicular to the donor - acceptor interface as presented in Fig. 4 (b). Here, the energy profile is given for three different cases: when the external electric field is applied perpendicular to the donor/acceptor interface (with positive values of ***F*** pointing from the donor to the acceptor phase), in the opposite direction (negative ***F*** values) and with no external electric field. Using our model we calculate the electron probability density 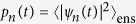 and the absolute e-h separation distance *d*_abs_(*t*) =〈∑_*n*_|*ψ*_*n*_(*t*)|^2^|***r***_***n***_ − ***r***_***D***_|〉_ens_. Here *ψ*_*n*_(*t*) is the electron amplitude on lattice site *n*, the sum runs over all system sites and 〈…〉_ens_ denotes ensemble averaging. We also introduce the average coherence radius of the electron 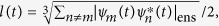 which denotes the linear extent of delocalization. Finally, we calculate the electon-hole drift separation distance 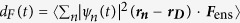 along the direction of the applied external electric field. Using the calculated drift distance we are also able to estimate the electron mobility 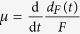.

## Additional Information

**How to cite this article**: Abramavicius, V. *et al.* Role of coherence and delocalization in photo-induced electron transfer at organic interfaces. *Sci. Rep.* **6**, 32914; doi: 10.1038/srep32914 (2016).

## Supplementary Material

Supplementary Information

## Figures and Tables

**Figure 1:**
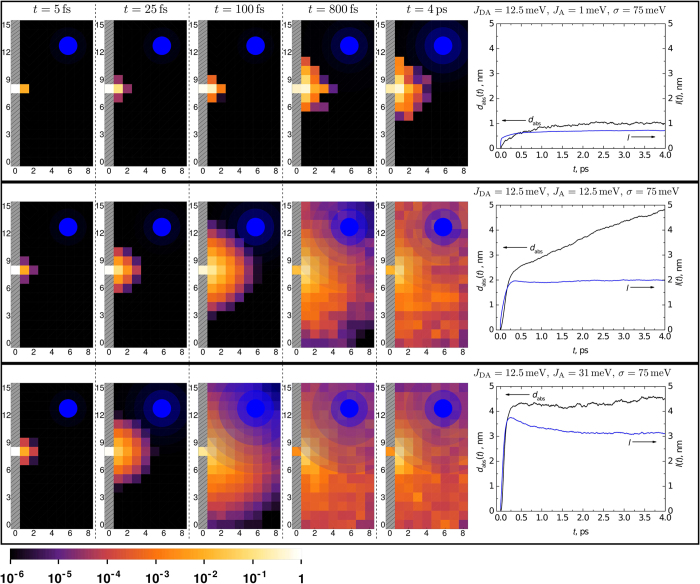
Ensemble-averaged evolution of electron probability density in the plane perpendicular to the donor (grey area) and acceptor (8 × 16 × 16 nm^3^ domain) interface at different times following photoexcitation for the indicated values of inter-acceptor coupling *J*_A_. The rightmost column shows the corresponding absolute charge separation distance *d*_abs_(*t*) (black traces) and delocalization radius *l*(*t*) (blue traces). Filled blue circles illustrate the extent of electron coherence at a given time. The color scale at the bottom describes the probability of finding the electron at the indicated distance from the interface.

**Figure 3:**
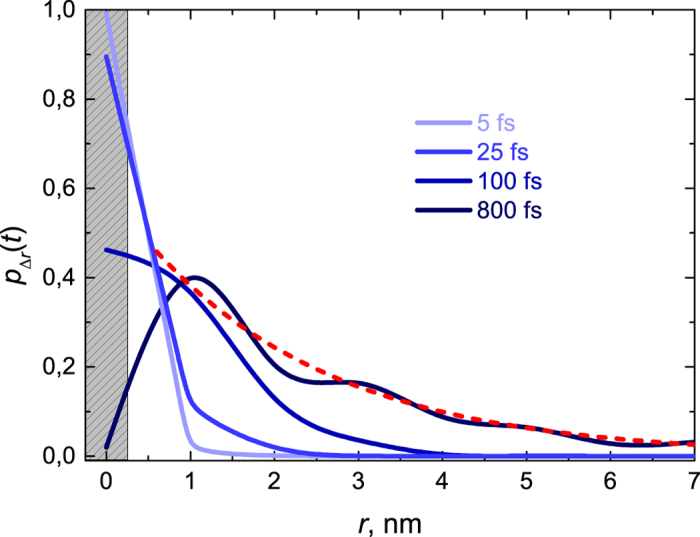
Radial distribution of (**e-h**) separation distance at early times. The shaded grey area indicates the position of the donor site. Dashed red line represents the exponential character of the e-h distance distribution after 800 fs.
