# Supplementary material for: Role of coherence and delocalization in photo-induced electron transfer at organic interfaces
Source: Sci Rep. 2016 Sep 8;6:32914. doi: 10.1038/srep32914 (PMC5015064; doi:10.1038/srep32914)
Supplement: Supplementary Information [file srep32914-s1.pdf]

Supplementary Information to

Role of coherence and delocalization in photo-induced electron transfer at organic interfaces

V. Abramavicius[1, 2], V. Pranckevičius[2], A. Melianas[3], O. Inganäs[3], V. Gulbinas[2], D. Abramavicius[1]

1- Vilnius University, Faculty of Physics, Department of Theoretical Physics, Saulėtekio 9, LT-10222 Vilnius

2- Center for Physical Sciences and Technology, Savanoriu 231, LT-02300 Vilnius, Lithuania

3- Biomolecular and organic electronics, Department of Physics, Chemistry and Biology (IFM), Linköping University, SE-581 83 Linköping, Sweden

Determination of the charge separation distance along the direction of the applied electric field

Fig. 1s shows the experimentally determined kinetic of the charge carrier drift distance. The initial part of the transient (0-3 ns) was determined by the TREFISH technique, whereas the latter part of the transient (3-10000 ns) was determined by a transient photocurrent measurement with a high load (integrated photocurrent). The photo-induced drop of the electric field was determined from the second harmonic intensity as described in [1s]:

$$\Delta F(t)/F = (I(t)/I_0)^{1/2} - 1 \qquad (1s)$$

where $I(t)$ and I_0 and are the second harmonic intensities with and without the excitation pulse respectively, and F is the internal electric field created by the applied voltage and the built-in field of the OSC, the latter was assumed to be 1V. Charge carrier drift distance distance $l(t)$ was determined form electric field dynamics by numerically solving eq. (2s) as described in [2s]:

$$\Delta F(t)_{norm} = \frac{2}{D^2}\int_0^t [D - l(t')]\frac{dl(t')}{dt'}dt' \qquad (2s)$$

where $DF(t)_{norm}$ is the electric field drop normalized to the voltage drop at full extraction (~10^4 ns in case of Fig. 1s) and D is the sample thickness. Charge carrier separation distance along the direction of the applied electric field equals to the sum of electron and hole drift distances, thus full extraction corresponds to D/2 = 35nm in Fig. 1s. The initial part of the transient in Fig. 1s is mainly dominated by electron motion, whereas the latter part of the transient is dominated by the motion of holes.

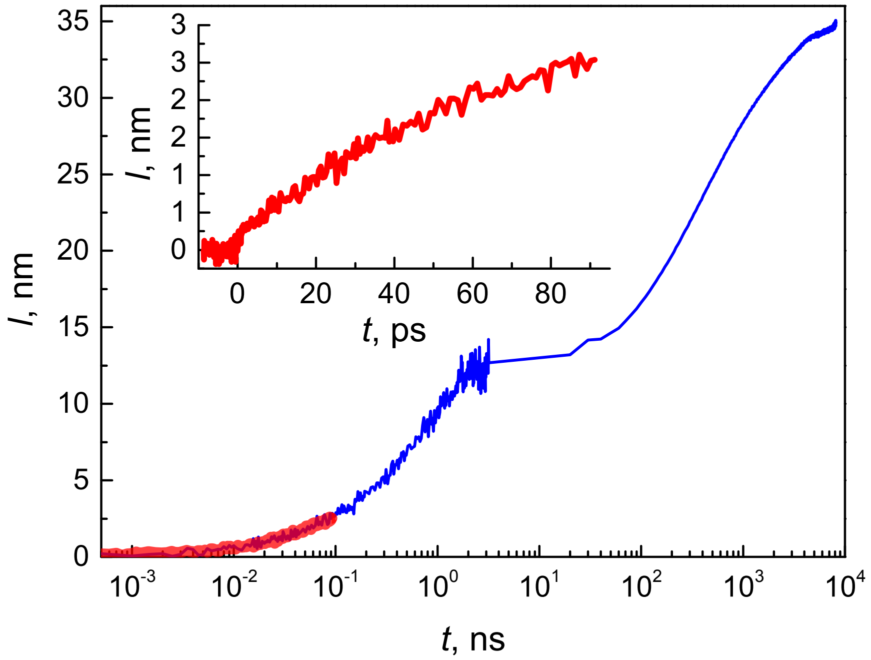

Figure 1s. Time dependence of the average carrier drift distance at an electric field of F = 5.7×10^5 V/cm. Inset shows the initial 100 ps of the kinetic which were measured with better accuracy for comparison with simulations.

Transient absorption spectroscopy

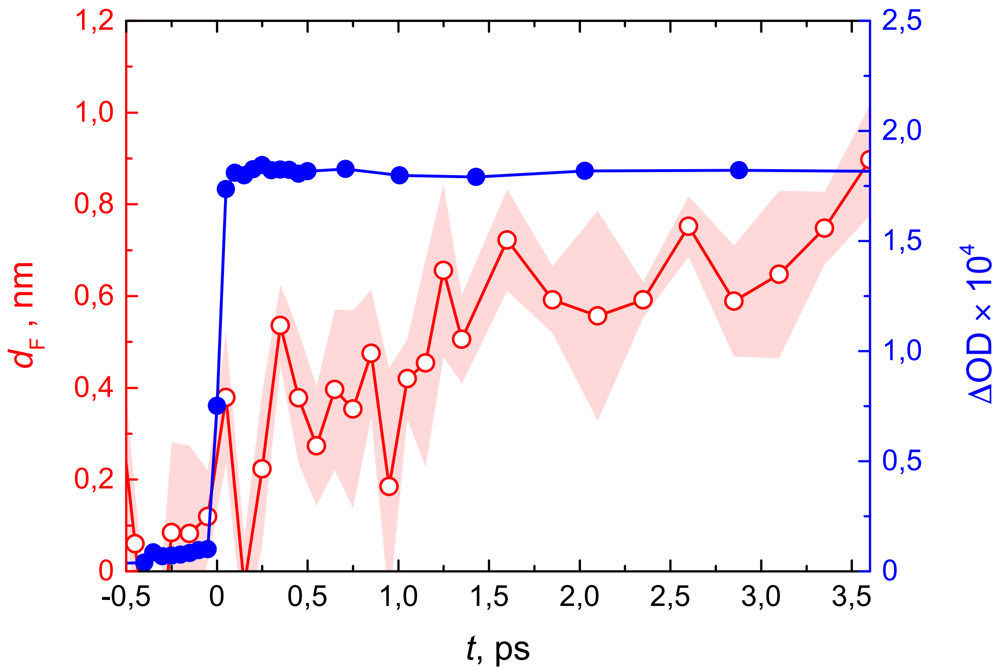

Figure 2s. Time dependence of the charge separation distance d_F as in Figure 2 of the main text (red) and a transient absorption (TA) kinetic (blue), indicating ultrafast <100fs photo-induced charge transfer with no signatures of delayed charge transfer due to exciton diffusion. Thus, the experimental EFISH data directly monitors the motion of the photo-induced electron away from the donor/acceptor interface. In TA the sample was excited at a pump fluence of 2.2×10^{12} photons/cm^2/pulse with 532nm photons and probed at 980nm which roughly corresponds to the center of a spectrally broad photoinduced absorption band.

Simulation parameters

The donor/acceptor interface is characterized by the lattice constant a, the molecular dimension parameter b (in the Coulomb potential expression), the interaction energies between sites J_{DA} and J_{A}, the donor site excitation energy ε_{D}, the disorder σ and the system – bath coupling strength λ. In addition, the overall size of the lattice could be variable due to the complexity of interfacial domains at hierarchical morphologies of the blend. We assume that differences in morphologies are mostly reflected by a variation of couplings between the acceptor sites. Simulation parameters were chosen as those typical for polymer-PCBM systems: driving force $\Delta E = 0.1$ eV for P3TI:PC$_{71}$BM, CT exciton binding energy $\varepsilon_{\mathrm{CT}} = -0.27$ eV, and the energetic disorder σ

of PCBM was set equal to $\sigma = 75$ meV, corresponding to the experimentally determined value in [3s] and J_{DA} is set equal to 12.5 meV leading to an electron transfer time of 100 fs, in agreement with experimental transient absorption data in Figure 2s. The lattice constant is equal to 1 nm. However, we point out that absolute values of these parameters, such as for example the energetic disorder of the PCBM phase, are model dependent. In order to determine the sensitivity of our simulations to different parameters, we have calculated the dynamics of the system by varying the parameters in the vicinity of the values given. For this investigation we used a smaller acceptor lattice of 8x8x8 sites. Results are presented in Figs. 3s – 6s. We find that the most important parameter governing the coherent dynamics (<500 fs) of the electron is the inter-acceptor coupling J_A. While the dynamics at later times (>500 fs) somewhat differ, they remain qualitatively the same within the range of parameter values reported by other studies. Hence, in later analysis we have kept all of the parameters fixed to those outlined above, except for the inter-acceptor coupling J_A, which was varied.

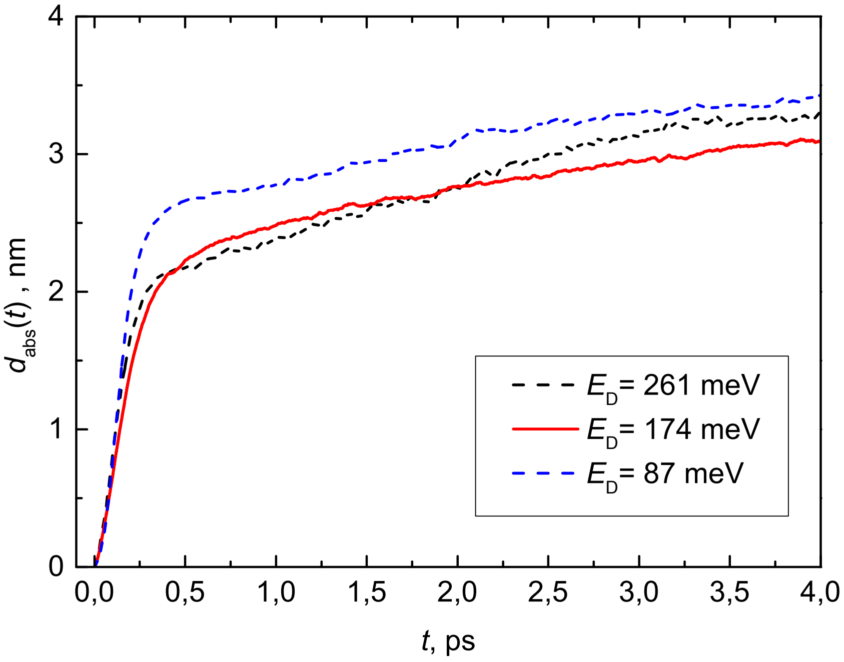

Figure 3s. Absolute charge separation distance $d_{abs}(t)$ for different values of donor excitation energy ε_D.

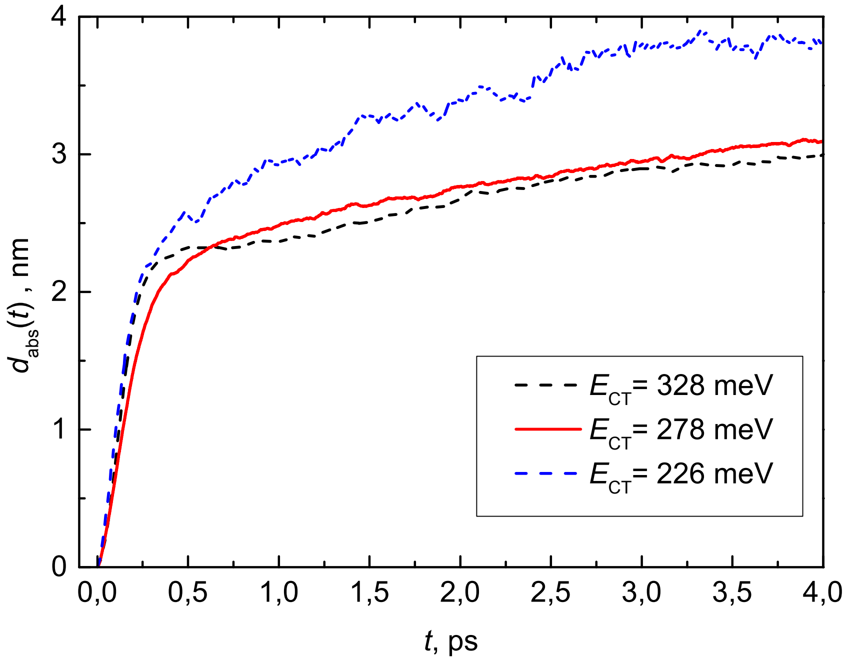

Figure 4s. Absolute charge separation distance $d_{\text{abs}}(t)$ for different values of CT state energy ε_{CT}.

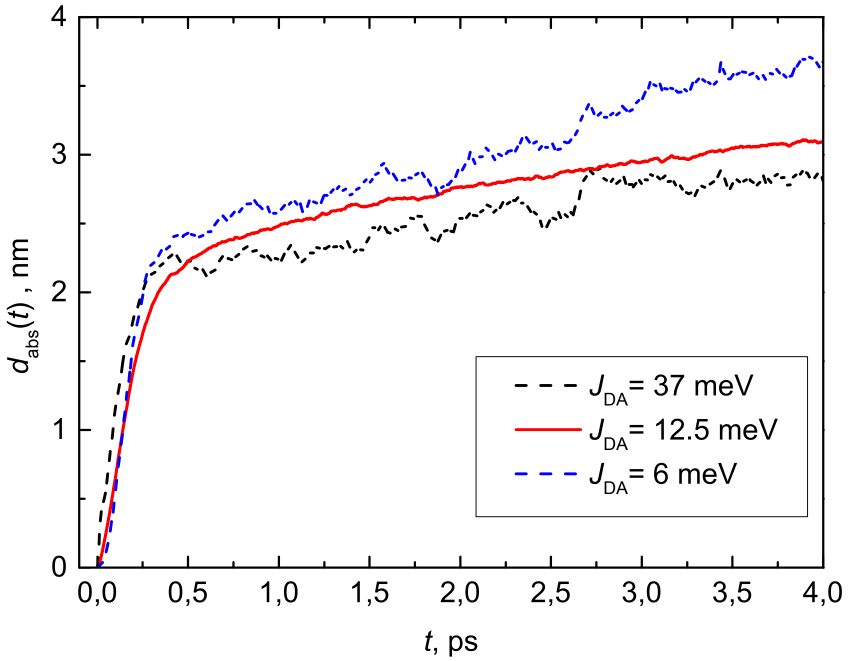

Figure 5s. Absolute charge separation distance $d_{\text{abs}}(t)$ for different values of donor-acceptor interaction energy J_{DA}.

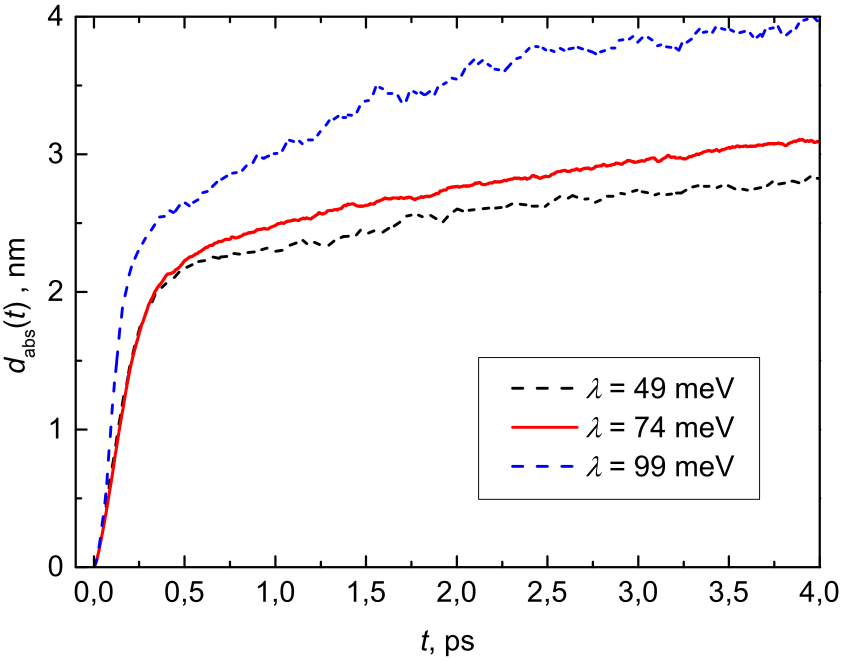

Figure 6s. Absolute charge separation distance $d_{\text{abs}}(t)$ for different values of system-bath interaction energy λ.

References:

[1s] Vithanage, D. A., et al. Visualizing charge separation in bulk heterojunction organic solar cells. *Nat. Commun.* **4,** 2334 (2013).

[2s] Pranculis, V., et al. Charge carrier generation and transport in different stoichiometry APFO3:PC61BM solar cells. *J. Am. Chem. Soc.* **136,** 11331-11338 (2014).

[3s] Mihailetchi, V. D., et al. Electron transport in a methanofullerene. *Adv. Funct. Mater*. **13,** 43-46 (2003).
